# Goistrat: gene-of-interest-based sample stratification for the evaluation of functional differences

**DOI:** 10.1186/s12859-025-06109-0

**Published:** 2025-04-05

**Authors:** Carlos Uziel Pérez Malla, Jessica Kalla, Andreas Tiefenbacher, Gabriel Wasinger, Kilian Kluge, Gerda Egger, Raheleh Sheibani-Tezerji

**Affiliations:** 1 Department of Pathology, Medical University of Vienna, Währinger Gürtel 18-20, Vienna, 1090 Austria; 2 Ludwig Boltzmann Institute Applied Diagnostics, Ludwig Boltzmann Gesellschaft, Währinger Gürtel 18-20, Vienna, 1090 Austria; 3 Comprehensive Cancer Center, Medical University of Vienna, Währinger Gürtel 18-20, Vienna, 1090 Austria; 4 Department of Biomedical Imaging and Image-Guided Therapy, Division of Nuclear Medicine, Medical University of Vienna, Währinger Gürtel 18-20, Vienna, 1090 Austria; 5 Christian Doppler Laboratory for Applied Metabolomics, Medical University of Vienna, Währinger Gürtel 18-20, Vienna, 1090 Austria

**Keywords:** Gene of interest, GSVA, GSEA, Node2Vec, FOLH1, PSMA, Prostate cancer

## Abstract

**Purpose:**

Understanding the impact of gene expression in pathological processes, such as carcinogenesis, is crucial for understanding the biology of cancer and advancing personalised medicine. Yet, current methods lack biologically-informed-omics approaches to stratify cancer patients effectively, limiting our ability to dissect the underlying molecular mechanisms.

**Results:**

To address this gap, we present a novel workflow for the stratification and further analysis of multi-omics samples with matched RNA-Seq data that relies on MSigDB curated gene sets, graph machine learning and ensemble clustering. We compared the performance of our workflow in the top 8 TCGA datasets and showed its clear superiority in separating samples for the study of biological differences. We also applied our workflow to analyse nearly a thousand prostate cancer samples, focusing on the varying expression of the *FOLH1* gene, and identified specific pathways such as the PI3K-AKT-mTOR gene sets as well as signatures linked to prostate tumour aggressiveness.

**Conclusion:**

Our comprehensive approach provides a novel tool to identify disease-relevant functions of genes of interest (GOI) in large datasets. This integrated approach offers a valuable framework for understanding the role of the expression variation of a GOI in complex diseases and for informing on targeted therapeutic strategies.

## Background

The heterogeneity of cancer unveils significant challenges to effective diagnosis, prognosis and treatment. Traditional approaches that treat cancer as if it were a uniform disease frequently fail to capture the complexities and variety that exist among patients. Patient stratification, which splits patients into categories based on molecular, genetic or clinical characteristics, has emerged as a significant approach for improving cancer prognosis and diagnostic accuracy. Stratification not only improves the understanding of tumour biology, but it also makes it easier to identify specific biomarkers, resulting in more personalised and effective therapeutics.

One of the pioneering studies in patient stratification was conducted by Perou et al. in 2002, who used gene expression profiling to classify breast cancer into distinct molecular subtypes [1]. According to this classification, there are significant differences in prognosis and responsiveness to treatment among the subtypes, suggesting that stratification could lead to more tailored and effective treatment strategies for breast cancer patients [1]. Similarly, the work by Parker et al. in 2009 introduced a supervised risk predictor model that stratified breast cancer patients into intrinsic subtypes, further refining prognostic assessments and guiding treatment decisions [2].

Stratification has also played a pivotal role in identifying and validating biomarkers for cancer diagnosis and prognosis. For instance, the study on pembrolizumab treatment in multiple cancer types identified a gene expression profile associated with response to the drug, providing a foundation for personalised immunotherapy approaches [3]. Such insights are invaluable for developing diagnostic tools that are more sensitive and specific to particular cancer subtypes, thereby improving early detection and outcome prediction.

Another common strategy to explore molecular differences or biological characteristics linked to gene expression changes is to separate patients or tissue samples based on the expression levels of a specific gene of interest (GOI). This often involves ranking samples by normalized RNA expression of the GOI and designating the top and bottom percentiles as high and low expression groups, respectively [4]. However, this approach can be limiting as it often overlooks the distribution of RNA expression values across the entire sample set, leading to potentially arbitrary groupings. More informed methods incorporate additional variables such as disease-free survival or time to recurrence in cancer-associated analyses, allowing samples to be divided at points where survival differences are most pronounced [5, 6]. Alternatively, other statistical methods might prove useful, such as fitting two Gaussian models to the RNA expression data and identifying their intersection between both distributions to separate samples into two groups [7].

Despite the utility of these approaches, none focuses on stratification of patient samples based on the activity of biological processes and enriched functions. In other words, they do not take the functional profile of each generated group into account to determine the optimal separation of samples. This is key to better understand how varying expression levels of the GOI impact biological processes involved in disease and thus better elucidate the biological role of the GOI.

**Fig. 1:**
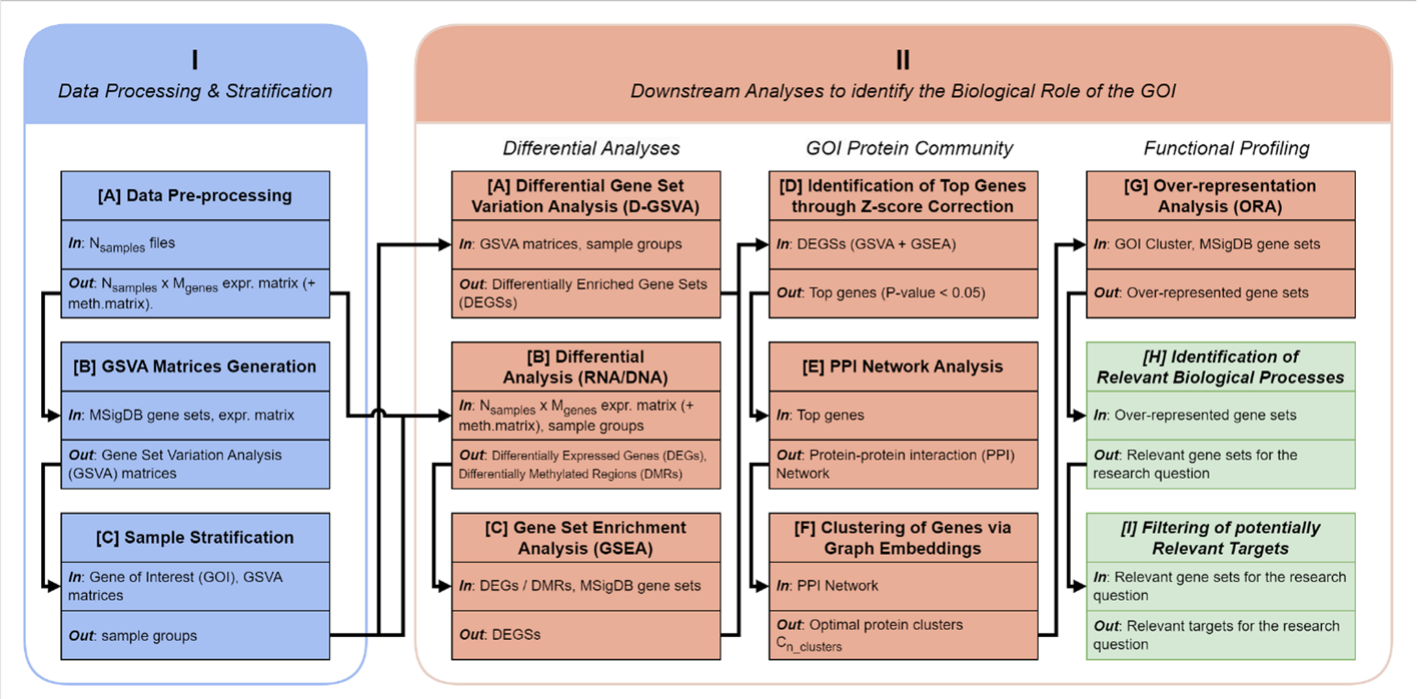
**Workflow diagram**. The workflow consists of two main phases. Phase I is the sample stratification that maximises the functional differences between low- and high-expressing RNA groups of a GOI. Phase II groups downstream analyses to better understand the biological role of the GOI, and includes differential analyses of the stratified samples, identification of the GOI protein community, and the functional profiling of the protein community. Phase II also includes two additional steps at the end, coloured in green, and meant to be performed by expert biologists using the data generated by the workflow

In response, we propose GoiStrat, a workflow including a novel approach for gene-level sample stratification that maximises the functional differences between low- and high-expressing samples, and downstream analyses to elucidate the functional profile of the GOI (Fig. 1). Our stratification algorithm relies on a functional score derived from the Gene Set Variation Analysis (GSVA) algorithm [8] using Molecular Signatures Database (MSigDB) gene sets [9], whereas our downstream analyses include gene set level differential analyses, unsupervised machine learning with Node2Vec [10] and ensemble clustering applied on PPI networks.

In this paper, we showcase the ability of GoiStrat to stratify samples across several cancer types and GOIs and show its stratification superiority as compared to the top/bottom approach.

Later, we applied the whole workflow specifically to prostate cancer patients using the *FOLH1* gene, which encodes the Prostate Specific Membrane Antigen (PSMA) protein, a clinically relevant biomarker for prostate cancer (PCa). According to GLOBOCAN 2020, PCa is the second most frequent cancer in men globally and ranks fifth in terms of cancer-related fatalities. Despite the establishment of multiple molecular and imaging biomarkers for tumour aggressiveness and diagnosis, their precision still needs improvement [11]. PSMA is a promising biomarker of growing interest, with expression levels correlated with PCa progression and poorer prognosis [12]. It is already used in positron emission tomography/computed tomography (e.g., 68Ga PSMA PET/CT) and radionuclide therapy (177Lu PSMA) [13, 14]. However, the biological role of PSMA in PCa is not yet well understood, despite recent insights [15]. Through our approach, we obtained genes and gene sets associated with known biological processes linked to *FOLH1* in PCa, such as increased tumour aggressiveness and poor prognosis, demonstrating the efficacy of our method.

## Methods

### Biologically-informed sample stratification

Phase I of the GoiStrat workflow represents a novel approach to stratify samples based on the functional differences between high and low RNA expression groups of a GOI (Fig. 1). It requires a pre-defined GOI (e.g. *BRCA1*) and a gene expression matrix (i.e. as obtained from an RNA-Seq experiment). Therefore, the workflow is applicable to any GOI, as long as the gene expression data is available. For example, for multi-omics datasets, RNA-Seq data can be used for sample stratification, while the other data modalities (e.g. DNA methylation, proteomics) can be used for differential analyses.

We represent functional information with GSVA matrices [8], calculated from the input gene expression matrix and all the collections in the MSigDB database [9] (i.e. C1-8 and H), resulting in a total of 9 GSVA matrices. GSVA transforms a gene expression matrix (i.e. a gene counts matrix of $N_{genes}$ and $M_{samples}$) to a gene set matrix (i.e. an enrichment scores matrix of $N_{gene\_sets}$ and $M_{samples}$). Each enrichment score is calculated as the maximum deviation from zero of a weighted running sum statistic, normalised with the gene set size [8]. It is worth noting that input gene expression matrix should be pre-processed following common good practices. These include removing lowly expressed genes, problematic samples as well as any existing batch effects.

The stratification algorithm follows a sliding window approach, where samples are first ranked based on the GOI expression values after variance stabilizing transformation (VST) [16] and then iteratively assigned to low-, mid- (i.e. a buffer) and high-expression groups (see Algorithm 1). The minimum number of samples per group is a configurable parameter. By default, mid is set to 50% of all samples, whereas high and low have at least 10% of all samples each. The algorithm runs for multiple iterations, advancing one sample at a time. For example, at the first iteration, the first 10% of the ranked samples are assigned to the low group, the consecutive 50% to the mid group, and the remaining 40% to the high group. Conversely, in the last iteration the first 40% of samples are assigned to the low group, the following 50% to the mid group, and the remaining 10% to the high group.

**Algorithm 1:**
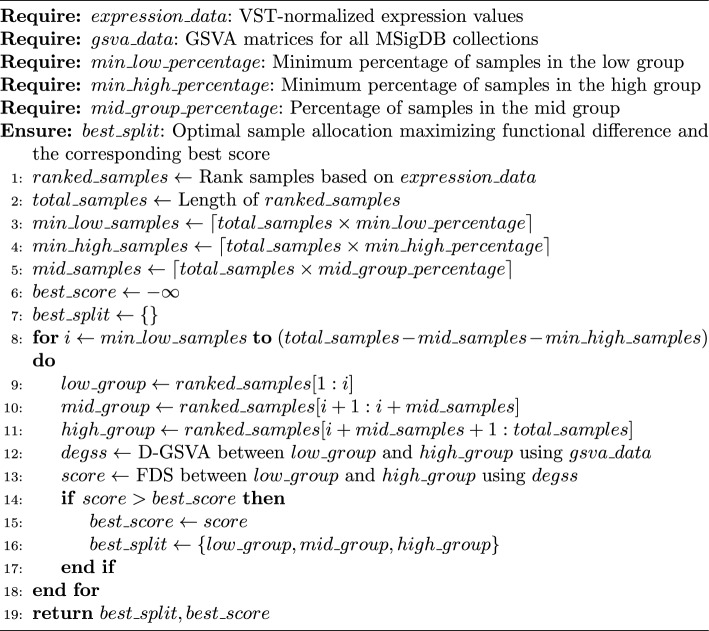
GoiStrat Stratification Algorithm

At each iteration of the algorithm, a functional difference score (FDS) is calculated based on statistically significant (P-value adjusted < 0.05) differentially enriched gene sets (DEGSs) from a differential gene set variation analysis (D-GSVA) based on GSVA matrices (see Algorithm 1). It is important to highlight that the differential analysis, as implemented in *limma* ([17]), can deal with the negative values present in the GSVA matrices. Furthermore, in the context of this analysis, log$_2$ fold changes are the estimated coefficients of the linear model fitted with *limma*. FDS is a continuous value representing the biological differences between two groups of samples (i.e. high vs low), with higher values indicating bigger differences. It is calculated per gene set collection, and is defined as the root-mean-square of the DEGSs’ log$_2$ fold changes multiplied by the DEGSs ratio (i.e. the number of DEGSs divided by the total number of gene sets in that collection). The final FDS of the iteration is the median of all collections’ scores (see Eq. 1). Finally, the optimal and final sample distribution is in the iteration with the maximum FDS.

1 $$\begin{aligned} FDS = \text {median} \left( \frac{n}{N} \times \sqrt{\frac{1}{n} \sum _{i=1}^{n} (\log _2 FC_i)^2}\right) \end{aligned}$$,

where:

- $\log _2 FC_i$ is the log$_2$ fold change of the *i*-th DEGS.
- *n* is the number of DEGSs.
- *N* is the total number of gene sets in the collection.
- $\text {median}$ is the median function applied over all MSigDB collections.

It is worth mentioning that the search performed by the algorithm is not exhaustive if run only once, due to the pre-defined minimum number of samples per group. We deemed these initial conditions necessary in order to reduce the computational cost of the algorithm and avoid wasting resources on extreme group combinations that might be statistically undesirable (i.e. where groups are too small or have too drastic differences in the number of samples). Therefore, for an exhaustive search, the algorithm would need to be run multiple times with different initial conditions. Fortunately, the computational cost of the algorithm scales approximately linear with respect to the number of samples and number of gene sets, with complexity $O(n \cdot m)$. Moreover, each iteration of the algorithm can be run independently in parallel, for a total of $(N_{total} - N_{low} - N_{high} - N_{mid})$ iterations per MSigDB category.

### Differential analyses

Phase II of the GoiStrat workflow starts with differential analysis between the high- and low-expression groups obtained from the stratification algorithm in phase I (Fig. 1). The workflow currently supports differential gene expression [16], differential methylation [18, 19] and differential enrichment (i.e. GSVA-based) [8] analyses, though other types of analyses, such as differential protein expression, could be easily integrated.

The main goal of these analyses is to identify the most relevant biological processes, represented by gene sets, associated with varying expression levels of the GOI. Each analysis type yields a different collection of gene sets, either directly via the differential gene set variation analysis (D-GSVA), or in a two-step process by first identifying differentially expressed genes (DEGs) or differentially methylated regions (DMRs) and then performing Gene Set Enrichment Analysis (GSEA) [20]. In the general case, differential analyses involve fitting a linear model and then estimating the P-values for each coefficient (e.g. gene, gene set). Then, these P-values are adjusted for multiple hypothesis testing by applying for example the Benjamini-Hochberg procedure ([21]). Details about the implementation of the differential analyses can be found in the GitHub repository. Finally, DEGs are filtered to be uniquely annotated to ENTREZID, and ranked by the log$_2$ fold change (LFC) statistic before GSEA. Similarly, prior to GSEA, DMRs are ranked using LFC or methylation differences, depending on which statistic is returned by the R/Bioconductor packages that process each methylation data type (e.g. microarray, WGBS).

We use the term up-regulated to refer to gene sets with a positive $log_{2}$ fold change (i.e. obtained from D-GSVA) or a positive enrichment score (i.e. obtained from GSEA), whereas down-regulated refers to gene sets with negative values in those metrics. Positive values indicate a higher activity of said gene set in the test group as compared to the control group, negative values indicate the opposite, and near-zero values indicate no activity differences.

### Identifying the protein community of the GOI

Continuing with phase II of the GoiStrat workflow, all the up-regulated (i.e. higher activity in high group) and down-regulated (i.e. lower activity in high group) DEGSs are collected from the previous step and combined (i.e. set union) to extract the genes most likely to be associated to differences in the expression of the GOI. More concretely, the union of DEGSs from all sources (e.g. D-GSVA, RNA-Seq GSEA, DNA Methylation GSEA) is computed for each MSigDB category, while keeping up-regulated and down-regulated gene sets separately. Then, Z-score normalised relative gene occurrence statistics (i.e. percentage of DEGSs a gene appears in) is calculated by bootstrapping random gene sets for a given MSigDB collection. Specifically, for any given collection, the relative gene occurrence of each gene within randomly selected *n* gene sets is computed, where *n* is the original number of DEGSs identified in that collection. The mean and standard deviation of this statistic is computed over 512 iterations, and then used to produce the Z-score normalised gene occurrence statistics of the identified DEGSs (see Eq. 2). Finally, the Z-scores are averaged over all collections, weighted by the DEGSs ratio (i.e. number of DEGSs divided by the total number of gene sets in that collection) (see Eq. 3), and the corresponding P-values are computed (see Eq. 4). All genes with a P-value below 0.05 are considered for the next step.

2 $$\begin{aligned} Z_{ij}&= \frac{r_{ij} - \mu _{R_{ij}}}{\sigma _{R_{ij}}} \end{aligned}$$

3 $$\begin{aligned} Z_{\text {final}, i}&= \frac{\sum _{j=1}^{m} w_j Z_{ij}}{\sum _{j=1}^{m} w_j} \end{aligned}$$

4 $$\begin{aligned} P_i&= 2 \left( 1 - \Phi \left( \left| Z_{\text {final}, i} \right| \right) \right) \end{aligned}$$,

where:

- $Z_{ij}$ is the Z-score normalized relative gene occurrence for gene *i* in collection *j*.
- $r_{ij}$ is the relative gene occurrence of gene *i* within DEGSs in collection *j*. It is calculated as $\frac{n_{ij}}{n_{j}}$, where $n_{ij}$ is the number of gene sets in collection *j* that gene *i* appears in and $n_{j}$ is the number of DEGSs found in collection *j*.
- $\mu _{R_{ij}}$ is the mean relative gene occurrence of gene *i* within randomly selected *n* gene sets in collection *j* computed over 512 iterations.
- $\sigma _{R_{ij}}$ is the standard deviation of the relative gene occurrence of gene *i* within randomly selected *n* gene sets in collection *j* computed over 512 iterations.
- $Z_{\text {final}, i}$ is the final Z-score for gene *i*.
- $w_j$ is the weight for collection *j*, calculated as $\frac{n_j}{N_j}$, where $n_j$ is the number of DEGSs found in collection *j* and $N_j$ is the total number of gene sets in collection *j*.
- *m* is the total number of collections.
- $P_i$ is the P-value for the final Z-score of gene *i*.
- $\Phi$ is the cumulative distribution function of the standard normal distribution.

The top genes identified within the DEGSs are used to build the protein-protein interaction (PPI) network, built separately for up-regulated and down-regulated DEGSs. To generate the PPI network, STRINGDB [22] is queried with the top genes to obtain the functional protein interactions with an interaction score bigger than 500, which is considered to represent medium-high confidence. Then, only the giant component of the graph (i.e. the largest set of connected nodes) is kept by removing all isolated nodes and any smaller components.

Next, Node2Vec is employed [10] to generate embeddings of the PPI network nodes. Node2Vec is a machine learning algorithm that generates low-dimensional vector representations (i.e. embeddings) of the nodes in a graph by generating random walks to train a skip-gram model [10]. Given a pair of nodes, their similarity can be determined by applying a similarity or distance function (e.g. euclidean distance, cosine similarity) to their embeddings. Node2Vec has multiple hyperparameters that can be tuned to adjust the model’s behaviour, which are however not trivial to test due to the unsupervised nature of the model. Relevant parameters include *p* and *q*, which control what kind of similarity the model is trained for (i.e. homophily vs structural equivalence), as well as the embeddings dimension, the number of random walks and their length. The *p* (return) and *q* (in-out) parameters are particularly of interest. If a value of *q* is set to be bigger than that of *p*, the model will learn structural similarity (i.e. similar nodes will be similarly connected, such as hub nodes), whereas in the opposite case it will learn homophily (i.e. similar nodes will belong to the same network communities) [10]. In order to circumvent the incompleteness of the human interactome, we set $p=1$ and $q=0.5$ to find communities of proteins.

The embeddings generated by Node2Vec are employed to group all nodes from the PPI network (i.e. proteins) using ensemble clustering [23] (see Algorithm 2), with the ultimate goal of identifying the protein cluster where the GOI resides. First, DBSCAN [24] estimates the initial number of clusters to avoid setting it as a tunable parameter and thus reduce complexity. Next, 128 weak clustering models (i.e. KMeans) are fit to generate the clustering similarity matrix [23]. This matrix essentially represents how often two nodes in the graph were clustered in the same group by each model, and thus give a sense of overall similarity. In other words, the more similar two nodes are in the graph, the more likely they will be clustered together by different models and thus their coefficient in the matrix will be higher. Finally, the final clustering results are obtained by feeding the clustering similarity matrix to a spectral clustering algorithm.

The clustering performance is measured using the silhouette score [25], and serves as a basis to tune the other hyperparameters of Node2Vec (see Algorithm 2). More concretely, grid search was employed to test many values combinations of embedding dimensions, walk lengths and number of walks. The silhouette score is a common metric to evaluate clustering methods, from which we derived the per-sample silhouette score, average silhouette score and the percentage of samples within each cluster with their silhouette score above the average score. The last metric served to discard clustering results if the percentage of any cluster was zero, and the average score was used to rank the remaining grid search iterations. Finally, the optimal Node2Vec hyper-parameter combination was the one that achieved the highest average silhouette score.

**Algorithm 2:**
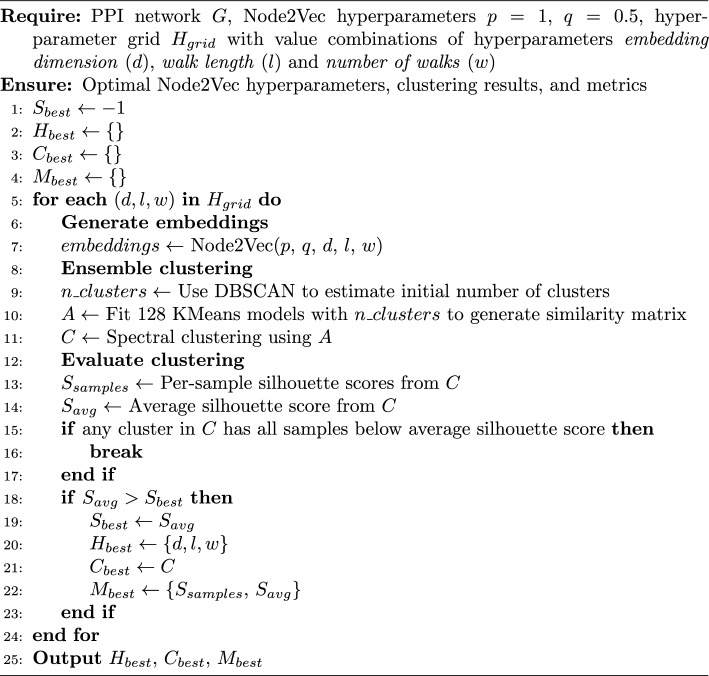
Node2Vec and ensemble clustering for PPI network

Once the optimal clustering results have been obtained, the protein cluster containing the GOI is identified and extracted, to be used in the next step.

### Functional profiling of the protein community

The last step of the GoiStrat workflow phase II carries out an over-representation analysis (ORA) in the previously-identified GOI cluster (i.e. the GOI protein community). By focusing only on the GOI cluster, we increase the chances that any functional gene sets or pathways found are specific to the GOI. More concretely, ORA is based on a hypergeometric test [26, 27] where the interesting genes are the ones coding for the proteins present in the GOI protein community, the background genes are all genes tested for differential expression (i.e. all genes for which enough transcriptional information was available and were thus not discarded) and the gene sets of interest are those in all the MSigDB collections (see Eq. 5).

5 $$\begin{aligned} P(X \ge x) = 1 - P(X \le x - 1) = 1 - \sum _{i=0}^{x-1} \frac{\left( {\begin{array}{c}M\\ i\end{array}}\right) \left( {\begin{array}{c}N-M\\ n-i\end{array}}\right) }{\left( {\begin{array}{c}N\\ n\end{array}}\right) } \end{aligned}$$,

where:

- $N$ is the number of background genes.
- $n$ is the number of interesting genes.
- $M$ is the number of genes that are annotated to a particular gene set $S$.
- $x$ is the number of interesting genes that are annotated to $S$.
- $$\left( {\begin{array}{c}M\\ i\end{array}}\right)$$ is the number of ways to choose $i$ genes from the $M$ genes in the gene set $S$.
- $$\left( {\begin{array}{c}N-M\\ n-i\end{array}}\right)$$ is the number of ways to choose $n-i$ genes not in the gene set $S$.
- $$\left( {\begin{array}{c}N\\ n\end{array}}\right)$$ is the total number of samples of size $n$ that can be taken from a population of size $N$.

The ORA results represent the final output of the GoiStrat workflow phase II, meant to be further examined by expert biologists.

### Comparison of GoiStrat with other methods

We compared the stratification ability of GoiStrat (i.e. phase II of the workflow) with the top/bottom approach based on quantiles using weighted FDS. To obtain the groups of the alternative approach, we ranked all samples based on the VST-normalised RNA expression of the GOI and then built the low group with the bottom quantile, the high group with the top quantile and the mid group with the rest of samples. We then calculated the FDS (see Eq. 1) between the high and low groups for each split of each method and weighted them based on the two sample t-test power, using an effect size of $d=0.5$ and a significance level of $\alpha =0.05$. Weighted FDS ensured a fairer comparison between methods given their varying group sizes.

### FOLH1 use case in prostate cancer

We based our case study on three distinct datasets, namely the Prostate Cancer Transcriptome Atlas (PCTA) [28], the Cancer Genome Atlas Prostate Adenocarcinoma (TCGA-PRAD) [29], and the West Coast Prostate Cancer Dream Team - Metastatic Castration Resistant Prostate Cancer (WCDT-MCRPC) [30, 31].

**Fig. 2:**
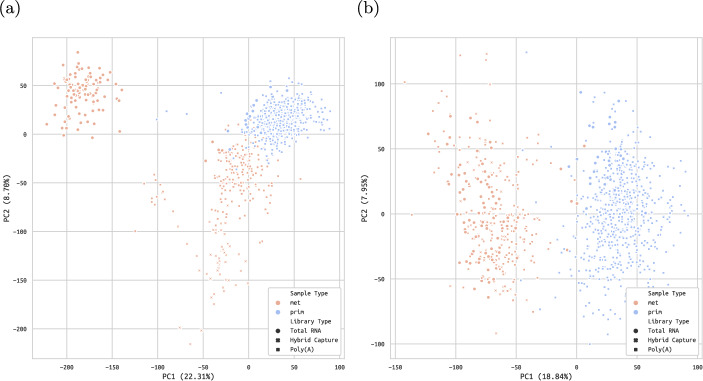
**Data processing**. PCA of PCTA-WCDT samples using (a) VST-normalised counts and (b) batch-corrected VST-normalised counts. See source data in Additional file 1

PCTA is a curated collection of 11 datasets comprised of PCa normal, primary and metastatic tissue RNA-Seq samples (Fig. 2a). The largest of these datasets include TCGA-PRAD, GTEX [32], GSE120741 [33] and phs000915 [34] (Supplementary figure B1a). We downloaded the raw read counts for all samples, as well as the clinical metadata, from the downloads section of the PCTA website (https://www.pcaprofilertest.tk). We then discarded all duplicated sample IDs: if a sample had a replicate with identical metadata except the sequencing library, we kept the replicates sequenced using the PolyA library. Most samples in PCTA used PolyA, so this decision helped minimise library heterogeneity. If all sample replicates had identical metadata, we kept the first and discarded the rest. After the removal of replicates, there were 662 primary and 237 metastatic castration resistant tumour samples left in our filtered version of PCTA.

TCGA-PRAD is part of the cancer genome atlas and offers a collection of 500 primary PCa cases. For most samples, different data modalities are included, such as transcriptome profiling, genome sequencing and DNA methylation. We downloaded the raw STAR read counts (RNA-Seq), the *.idat* files (methylation array) as well as the clinical metadata of matched samples from the GDC data portal. After minimal data cleaning, the resulting number of matched RNA-Seq and methylation array samples was 497.

WCDT-MCRPC is a collection of 100 metastatic castration resistant tumour samples. For this study, we selected 99 samples that underwent both transcriptome profiling (RNA-Seq) and whole genome bisulfite sequencing (WGBS). Metastatic samples were predominantly extracted from lymph nodes and bone tissues. We manually downloaded the raw STAR read counts (RNA-Seq) from the GDC data portal and the raw SRA files (WGBS) from the database of Genotypes and Phenotypes (dbGaP) (study accession: phs001648.v2.p1). Additionally, clinical annotations were obtained both from dbGaP and from the NCBI SRA Run Selector website. Finally, we obtained the methylation coverage files from the raw WGBS samples using our in-house pipelines, after mapping the adapter-trimmed samples to the human genome (version GRCh38).

In order to use all available samples, we joined PCTA and WCDT RNA-Seq samples in a single dataset, which we named PCTA-WCDT. We corrected for batch effects due to different sequencing libraries (Fig. 2b, Additional file 1 - Table 1A), estimated via empirical Bayes after fitting the gene counts with a negative binomial regression model [35]. We also discarded the healthy (i.e. normal) control samples, which were not within the scope of our investigation. Therefore, for the purposes of our analyses, PCTA-WCDT included 662 primary samples and 336 metastatic samples, with the latter having been predominantly extracted from lymph node (80), liver (53) and bone (50) tissues (Supplementary figure B1b).

We ran the GoiStrat workflow for *FOLH1* for all three datasets on the primary and metastatic samples separately, when available. RNA-Seq samples in each dataset were used to generate the GSVA matrices for sample stratification. In PCTA-WCDT, differential expression and differential gene set enrichment analysis were performed on the RNA-Seq samples. On the other hand, DNA methylation samples from TCGA-PRAD and WCDT-MCRPC were employed for differential methylation analyses. Sample stratification and differential analyses were performed independently for each dataset and sample type. The resulting DEGSs were combined for the identification of the top genes in the second step of the phase II of the workflow. Table 1 summarises each dataset used in this analysis and its purpose.

**Table 1:** Summary of the datasets used in the FOLH1 case study, including their composition, number of samples, data type and purpose

**Dataset**	**Components**	**Samples**	**Type**	**Purpose**
PCTA-WCDT	PCTA (TCGA-PRAD + other 10 datasets) + WCDT-MCRPC	998 (662 primary and 336 metastatic)	RNA-Seq	Differential expression and differential gene set enrichment analyses.
TCGA-PRAD	TCGA-PRAD	497	Paired RNA-Seq and Methylation Array	Differential methylation analysis.
WCDT-MCRPC	WCDT-MCRPC	99	Paired RNA-Seq and WGBS	Differential methylation analysis.

## Results

### GoiStrat shows superior performance in maximising functional differences between high and low groups

We compared the stratification performance of GoiStrat, corresponding to phase I of the entire workflow (Fig. 1), with different quantiles of the top/bottom split approach on 9 distinct RNA-Seq datasets. These include the 8 TCGA datasets [36] with the highest number of RNA-Seq primary samples, as well as PCTA-WCDT, our collection of more than 10 PCa datasets that combines PCTA [28] and WCDT-MCRPC [30, 31]. The TCGA datasets we selected were TCGA-BRCA (breast cancer) [37], TCGA-LUAD (lung adenocarcinoma) [38], TCGA-THCA (thyroid carcinoma) [39], TCGA-UCEC (uterine corpus endometrial carcinoma) [40], TCGA-LUSC (lung squamous cell carcinoma) [41], TCGA-KIRC (kidney renal clear cell carcinoma) [42], TCGA-HNSC (head-neck squamous cell carcinoma) [43], and TCGA-LGG (lower grade glioma) [44]. For each TCGA dataset, we performed basic pre-processing: removal of duplicated samples, removal of healthy tissue samples and filtering of transcripts to only include those with unique ENTREZID identifiers (Workflow step I.A.).

We chose the GOI for each dataset based on their relevance in the literature and their potential as diagnostic and/or prognostic biomarkers. For TCGA-BRCA, we chose *BRCA1*, a well-known gene in breast cancer involved in DNA repair whose over-expression is correlated with worse survival [45]. For TCGA-LUAD we selected *NKX2-1*, whose downregulation has been shown to be linked with metastatic growth [46]. We picked *HMGA2* for TCGA-THCA, whose up-regulation is associated with increased malignant growth and tumour aggressiveness of thyroid tumours [47]. *PIK3CA* was the GOI for TCGA-UCEC, whose mutations are common in endometrial cancer [48] and are associated with poor survival [49]. For TCGA-LUSC we designated *SOX2*, which plays a key role in promoting the disease from different cells of origin [50]. For TCGA-KIRC we chose *CA9* due to its diagnostic and prognostic value [51]. For TCGA-HNSC we selected *TP63*, which is overexpressed in primary tumours but often downregulated in advanced stages [52]. For TCGA-LGG we picked *IDH1*, whose mutations are associated with positive prognosis [53]. Finally, for our PCa case study (i.e. PCTA-WCDT) we chose *FOLH1*, a key biomarker in PCa already in use in the clinic but whose function and role in PCa is not yet fully understood [12].

For our methods comparison, we grouped samples into low-, mid- and high-expressing GOI groups using GoiStrat with default parameters (i.e. a minimum of 50% of samples in the mid group) and the top/bottom split approach with different top/bottom quantiles (i.e. 10%, 15%, 20%, 25% and 30%) (Workflow steps I.B. and I.C., Supplementary table A1). We compared each method in their ability to separate samples such that the functional differences between high and low groups were maximised, for which we used our Functional Difference Score (FDS). In order to show a fair comparison between methods, we weighted each score by the test power coefficient, which measures the likelihood of detecting differences between groups of samples as a function of the effect size to be detected, the sizes of each group of samples and a significance level (i.e. $\alpha$) (Supplementary table A2).

**Fig. 3:**
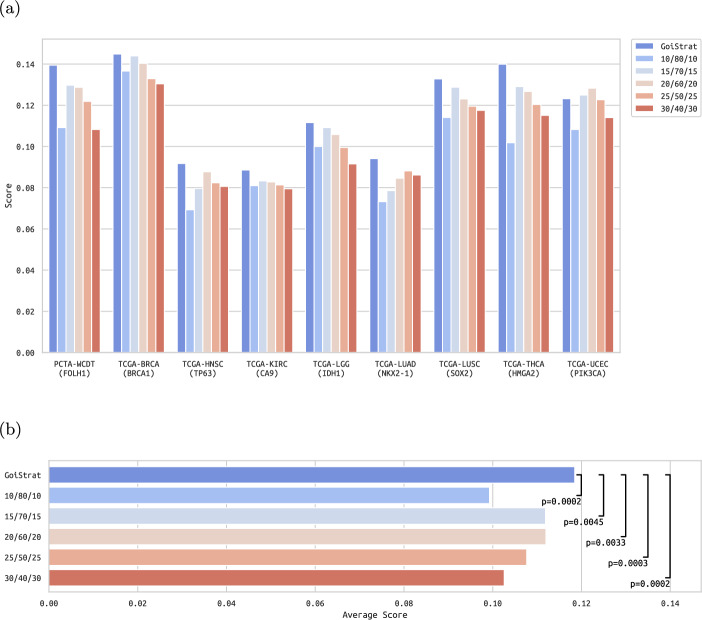
**Methods comparison**. (a) Our method is compared against multiple quantiles of the top/bottom split strategy in 9 datasets. GoiStrat outperforms the rest in 8 out of 9 of these datasets. The 15% quantile of the top/bottom split strategy ranks second place on average, whereas in most datasets higher quantiles are correlated with worse performance. (b) On average, the scores of GoiStrat are statistically significantly bigger than all variants of the top/bottom split strategy across all tested datasets

Our results showed that GoiStrat outperformed the top/bottom split approach in all datasets except one (Fig. 3a). Moreover, it showed a higher consistency in its efficacy between datasets as compared to the other methods. For example, in PCTA-WCDT, the 30% quantile performed the worst, close to the 10% quantile, whereas in TCGA-LUAD it was the second-best performer, way above the 10% quantile. Finally, we show that the average scores achieved by GoiStrat are statistically significantly superior to the top/bottom approach variants for all datasets (Fig. 3b). P-values were calculated using the one-tailed paired t-test, with the null hypothesis being that the average score of GoiStrat and each top/bottom approach is equal and the alternative hypothesis being that the average score of GoiStrat is bigger than the score achieved by each top/bottom approach.

These results demonstrate that, for this choice of datasets and GOIs, GoiStrat is a more robust and reliable method for GOI-based sample stratification, by iteratively finding the splitting point that maximises the functional differences between high and low groups. This first step is essential for downstream analyses, aimed at identifying relevant biological processes impacted by the GOI and thus support the better understanding of the biological role of the GOI in the context of the disease.

### FOLH1-based sample stratification maximises functional differences between high and low expressing samples

For the *FOLH1* case study, we used the RNA-Seq samples from PCTA-WCDT, as well as the RNA-Seq and matched DNA methylation samples from TCGA-PRAD and WCDT-MCRPC for a multi-omics approach.

In order to understand the biological role of *FOLH1* in PCa, we studied the differences between groups of samples with varying *FOLH1* RNA expression. More concretely, as part of phase I of the workflow, we employed a sliding window algorithm to assign samples to low, mid and high *FOLH1* expression groups at each iteration. Minimum percentage of samples per group were set as initial constraints (10%, 50% and 10%, respectively). By sliding a window of size n/2 (i.e. the mid group), we assigned different samples to each group at each iteration. For each iteration, we computed the FDS based on differentially enriched gene sets (DEGSs) mined from MSigDB obtained from a Differential Gene Set Variation Analysis (D-GSVA), which we used as a proxy to determine biological differences. It is worth noting that log$_2$ fold changes returned by D-GSVA are an approximation of the effect size as determined by *limma* after fitting a linear model to the gene counts. The maximum functional score determined the optimal sample stratification to be studied in further analyses (Workflow step I.B.).

We followed this strategy for primary and metastatic samples in the pre-processed PCTA-WCDT dataset (Workflow step I.A.), as detailed in the methods, thereby defining the *FOLH1* expression groups for each downstream analysis. From a total of 662 primary samples, 233 were assigned to the low group, 331 to the mid group and 98 to the high group (Supplementary figure B1c). Similarly, of 336 metastatic samples, 131 were allocated to the low group, 168 to the mid group and 37 to the high group (Supplementary figure B1d).

It is worth mentioning that while TCGA-PRAD and WCDT-MCRPC RNA-Seq samples are included in PCTA-WCDT, they were stratified separately for the downstream methylation analyses to only include the matched RNA-Seq samples. In other words, before performing differential methylation analyses, DNA methylation samples also had to be stratified into *FOLH1* groups, for which we used the matching RNA-Seq samples. Out of the 497 matched primary samples in TCGA-PRAD, 164 were assigned to the low group, 248 to the mid group and 85 to the high group (Additional file 1 - Table 1B). Similarly, the 99 metastatic samples in WCDT-MCRPC were divided as follows: 18 in the low group, 49 in the mid group and 32 in the high group (Additional file 1 - Table 1C).

### Differential analyses reveal specific functional differences between *FOLH1*groups

Next, we focused on curated gene sets to determine biological differences between the *FOLH1* expression groups. More concretely, we relied on MSigDB to extract gene sets from different collections. Our hypothesis was that by examining enrichment differences of these gene sets between *FOLH1* groups, we would be able to gain better insights into the biological role of *FOLH1* in PCa. All MSigDB collections (i.e. C1-C8 and H) were included in our analyses in order to capture every potentially relevant gene set *FOLH1* is involved in, regardless of the context. Since certain genes have been considerably studied more often than others, statistical corrections were also added to prevent bias towards frequently occurring genes caused by overlapping gene sets or differences in research effort. Given the vast number of gene sets in the MSigDB, we focused on the Hallmarks collection of MSigDB [54] for qualitative assessment of the results and to infer biological processes. This compilation encompasses curated gene sets based on the original C1-C8 collections and represents a good, refined summary of relevant biological functions.

We first obtained DEGSs through D-GSVA for primary and metastatic samples (Workflow step II.A., Supplementary table A3). D-GSVA was based on gene set enrichment score matrices obtained by transforming our gene counts matrices (i.e. transcripts x samples) into gene set enrichment scores matrices (i.e. gene sets x samples) for each MSigDB collection and for each sample type independently.

**Fig. 4:**
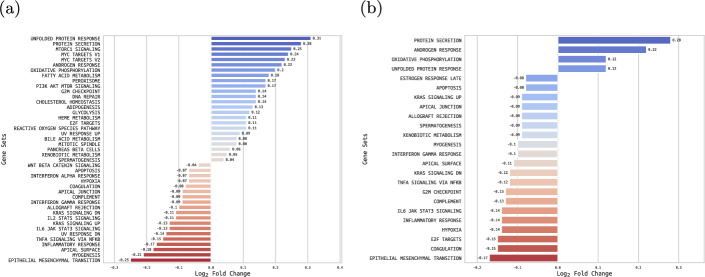
**D-GSVA Analysis on MSigDB Hallmarks Collection**. differentially enriched gene sets from D-GSVA on (a) primary and (b) metastatic samples

We identified some well-known functions previously linked to *FOLH1* including PI3K-AKT-mTOR signalling and mTORC1 signalling (Fig. 4a, Additional file 2 - Table 2A), which was up-regulated in *FOLH1* high samples in primary PCa [12, 55, 56]. Overall, metabolic pathways (e.g. fatty acid, bile acid) were up-regulated in the *FOLH1* high group, whereas inflammatory pathways including IL6-JAK-STAT3 signalling were down-regulated. Interestingly, loss of *STAT3* and induction of mTORC1 signalling was recently associated with more aggressive PCa in a transgenic mouse model [57]. In line with this, we found down-regulated IL6-JAK-STAT3 signalling in metastatic samples (Fig. 4b, Additional file 2 - Table 2B). Together, these findings suggest that *FOLH1* is closely linked with changes in metabolic and inflammatory pathways in primary and metastatic PCa.

In order to strengthen our analysis with a multi-omics approach, we also obtained enriched gene sets from Gene Set Enrichment Analysis (GSEA) on both unfiltered DEGs and differentially methylated regions (DMRs), again for both primary and metastatic samples (Workflow steps II.B. and II.C., Supplementary table A3). To determine the DMRs, we selected all genes that had at least one differentially methylated CpG site within the promoter region, and averaged the differential methylation metrics over CpG sites within individual promoters. It is worth mentioning that within the DEGs and DMRs in primary samples we identified *CYP27A1* (down-regulated and hyper-methylated) (Supplementary figure B2a, Additional file 2 - Table 2C), which is involved in cholesterol homeostasis and has been linked to worse PCa prognosis [58, 59]. Within the GSEA results from DEGs (Supplementary figure B2b, Additional file 2 - Table 2D) we again found up-regulated androgen response and down-regulated IL6-JAK-STAT3 signalling. The GSEA data from DMRs (Supplementary figure B2c, Additional file 2 - Table 2E), on the other hand, present a distinct picture, with IL6-JAK-STAT3 signalling significantly up-regulated. This asymmetric result could suggest that the RNA expression of some genes in that pathway are being suppressed by others that are hyper-methylated. In the tumour microenvironment, IL-6/JAK/STAT3 signalling acts to drive proliferation, survival, invasiveness, and metastasis of tumour cells, while strongly suppressing the antitumour immune response. Thus, treatments that target the IL6-JAK-STAT3 signalling pathway in patients with cancer are poised to provide therapeutic benefit by directly inhibiting tumour cell growth and by stimulating antitumour immunity [60]. In metastatic samples, the multi-omics approach (Supplementary figure B2d, Additional file 2 - Table 2F) identified the *MCU1* gene (down-regulated and hypo-methylated), which is believed to promote PCa tumourigenesis [61] and be linked to advanced PCa [62]. We also identified *LAMB3* (again down-regulated and hypo-methylated), which has been linked to the PI3K/AKT signalling pathway in metastatic pancreatic cancer [63]. The GSEA results from DEGs (Supplementary figure B2e, Additional file 2 - Table 2 G) showed a significantly down-regulated spermatogenesis, possibly a consequence of cancer therapies undergone by the patients with most aggressive cancers [64]. Finally, no statistically significant GSEA results were obtained from metastatic DMRs, suggesting the lack of overlap between the methylation signatures present in our data and known biological processes.

### Extracting the PSMA protein community by clustering the protein-protein interaction network of key genes

After identifying the functional differences between the individual *FOLH1* gene expression groups, we explored the relevance of genes within those gene sets. In other words, our next aim was to delineate which genes are most likely linked to *FOLH1* and tumour progression, by examining the occurrence of each gene within the obtained DEGSs.

We combined (i.e. outer join) the DEGSs from D-GSVA and GSEA analyses to build our collection of significantly deregulated gene sets and searched for the most important genes appearing within them (Workflow step II.D.). We calculated the relative gene occurrence for each gene within each MSigDB collection and performed z-score normalisation to ensure that over-abundant genes are not selected purely due to chance. Next, we averaged the z-score-corrected gene occurrences of the MSigDB collections and computed the P-value of the resulting z-scores. Finally, we selected all genes with a P-value below 0.05 as our potential targets to be studied further.

**Fig. 5:**
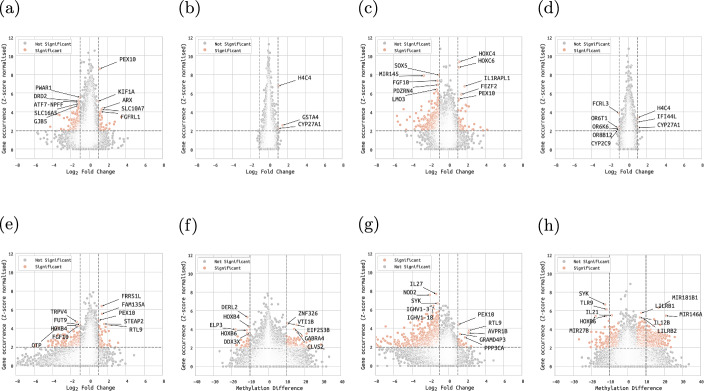
**Identification of top (i.e. most prevalent) genes extracted from DEGSs**. Relationship between z-score normalised occurrence of (a) DEGs in primary up-regulated gene sets, (b) DMRs in primary up-regulated gene sets, (c) DEGs in primary down-regulated gene sets, (d) DMRs in primary down-regulated gene sets, (e) DEGs in metastatic up-regulated gene sets, (f) DMRs in metastatic down-regulated gene sets, (g) DEGs in metastatic down-regulated gene sets and (h) DMRs in metastatic down-regulated gene sets

In primary samples, we identified a total of 7,833 and 5,991 significant genes in up-regulated and down-regulated gene sets, respectively. We also examined the relationship between the gene occurrence scores and the gene expression / gene methylation LFC scores. Among the DEGs and DMRs in up-regulated gene sets (Fig. 5a, 5b, Additional file 3 - Table 3A), we found down-regulated *DRD2*, known to be suppressed in advanced PCa [65]. Out of the DEGs and DMRs in down-regulated gene sets (Fig. 5c, 5d, Additional file 3 - Table 3B), we found up-regulated *HOXC4* and *HOXC6*, which are known PCa biomarkers [66]. Additionally, *SOX5* was down-regulated, which has been found to be a contributor to metastatic PCa [67].

In metastatic samples, a total of 5,690 and 5,809 genes were found to be statistically significant in up-regulated and down-regulated gene sets, respectively. We also studied the link between the gene occurrence scores, the gene expression LFC scores and the mean methylation differences. Considering the DEGs and DMRs in up-regulated gene sets (Fig. 5e, 5f, Additional file 3 - Table 3C), we obtained down-regulated *TRPV4*, in agreement with previous research [68]. Down-regulated *IL27*, a potential anti-tumour factor in PCa, was discovered in the DEGs and DMRs in down-regulated gene sets (Fig. 5g, 5h, Additional file 3 - Table 3D) [69].

In order to better understand the connection between our pre-selected targets and PSMA, we extracted the protein-protein interactions (PPI) between our potential targets by querying STRINGDB [22] (Workflow step II.E.).

We focused on PSMA and the community it belonged to, determined by clustering the PPI network nodes into multiple communities (Workflow step II.F.). We calculated embeddings (i.e. feature vectors) for each node in the network using the Node2Vec [10] machine learning algorithm. Then, we used ensemble clustering [23] to group the nodes based on their embeddings. Finally, we selected the cluster PSMA was assigned to for further analysis. In primary samples, PSMA communities were composed of 1,936 and 2,425 genes from up-regulated and down-regulated gene sets, respectively (Supplementary table A4). In metastatic samples, PSMA communities were composed of 936 and 783 genes from up-regulated and down-regulated gene sets, respectively (Supplementary table A4).

### Functional profiling of PSMA protein community

After defining the protein community around PSMA, we performed another functional analysis to determine its functional profile (Workflow step II.G.). However, because we were working with small sets of genes, we resorted to a simpler approach (as compared to GSEA): over-representation analysis.

**Fig. 6:**
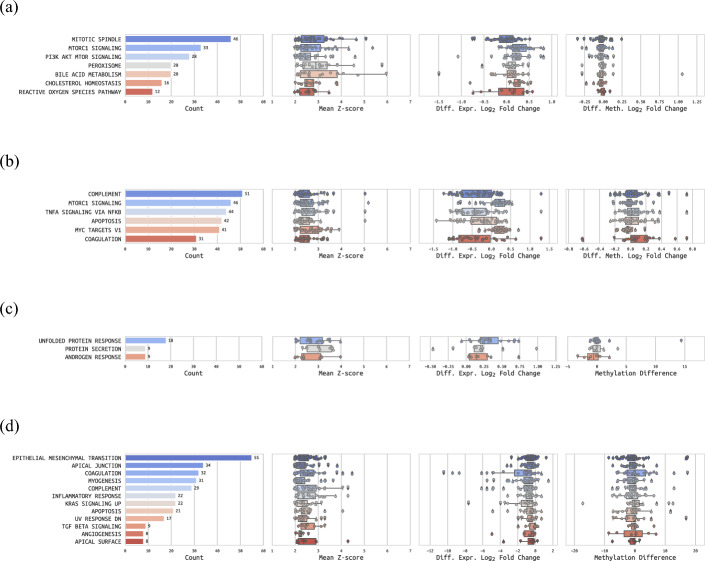
**Over-representation analysis on MSigDB Hallmark gene sets of the PSMA protein communities**. ORA results for (a) primary up-regulated gene sets, (b) primary down-regulated gene sets, (c) metastatic up-regulated gene sets and (d) metastatic down-regulated gene sets. The number of genes in each gene set is shown, as well as their mean z-score, their differential expression $log_{2}$ fold changes and their differential methylation $log_{2}$ fold changes (Prim. samples) or methylation differences (Met. samples)

In the up-regulated PSMA community of primary samples (Fig. 6a), we found the mTORC1 signalling as well as the PI3K-AKT-mTOR signalling pathways, which have already been linked to PSMA [12]. Additionally, we found the peroxisome gene set up-regulated, which is associated with lipid metabolism and potentially linked to cancer proliferation [70, 71]. In addition, cholesterol homeostasis was also among the results, which is negatively correlated to PCa prognosis, as discussed before. We also examined the metrics from the differential analyses (Fig. 6a), and identified *CYP27A1* (down-regulated, hyper-methylated), whose low expression is correlated with higher cholesterol and worse PCa prognosis [58].

In the down-regulated PSMA cluster of primary samples (Fig. 6b, Additional file 4 - Table 4A), we interestingly found mTORC1 signalling again, which could suggest differing behaviours of different sections of the pathways. Complement was also present, a gene set containing genes of the complement system, which can be pro- or anti- tumourigenic depending on the cancer type [72]. Next, we identified several pathways related to PCa progression, such as TNF-Alpha signalling via NF-$\kappa$B and MYC Targets V1. *NF-*$\kappa$*B* has been previously described as a contributing factor to PCa progression [73], whereas MYC Targets V1 is related to cell proliferation [74]. Not surprisingly, apoptosis is among these results, whose depletion leads to proliferation of cancer cells and can be an indicator of cancer aggressiveness. Coagulation alterations have also been hinted as a side-effect of cancer development, such as disseminated intravascular coagulation in PCa [75]. Surprisingly, among the most relevant genes within these gene sets (Fig. 6b, Additional file 4 - Table 4C), we identified *IGFBP6* (down-regulated), whose down-regulation was associated with lower cell proliferation in androgen-independent PCa cells in a past study [76]. This unexpected result would require further investigation that is out of the scope of this work. Another potentially relevant gene was *SERPING1* (down-regulated), which has been shown to allow for the distinction of higher risk PCa tumours [77].

In the up-regulated PSMA cluster of metastatic samples (Fig. 6c, Additional file 4 - Table 4B), we identified unfolded protein response, which cancer cells use as survival mechanism [78]. Additionally, androgen response was also present, which is in line with alterations of androgen signalling in castration-resistant metastatic PCa.

In the down-regulated PSMA cluster of metastatic samples (Fig. 6d, Additional file 4 - Table 4D), we observed coagulation, complement and apoptosis gene sets among the top results once again, same as in the down-regulated primary results, implying a link between PSMA expression and tumour aggressiveness in both primary and metastatic samples. Notably, the top result was the epithelial mesenchymal transition gene set, which has been suggested to increase the probability of cancer cells becoming metastatic by increasing cell mobility and apoptosis resistance [79]. It is worth mentioning that mesenchymal cells may also play a role in converting androgen-dependent PCa cells into androgen-independent cells, in a process that involves *TGF-*$\beta$, also present in these results. After inspecting the differential analysis metrics of the involved genes (Fig. 6d), we observed more than a dozen heavily down-regulated genes (LFC < -3). Of the top 6 down-regulated genes, *FGA*, *FGG*, *APOA1*, *HNF4A* and *SERPINA1* belonged to the coagulation gene set. While *SERPINA1* (hypo-methylated) promotes tumourigenesis in colorectal cancer [80], *HNF4A* (hyper-methylated) has been associated with tumour suppressor functions [81]. Another interesting gene was *VTN* (down-regulated, hyper-methylated), whose abundance in the blood serum is negatively correlated with PCa progression, and is thus expected to be less abundant in metastatic PCa as compared to primary PCa [82]. In addition, *MMP7* (down-regulated, hypo-methylated), together with *IL17*, also promotes PCa via epithelial-to-mesenchymal transition [83].

To conclude, the up-regulated and down-regulated pathways of each PSMA protein community showed a clear relationship between PSMA and tumourigenesis, both in primary and in metastatic samples.

## Discussion

Stratifying patients is a frequent approach in clinical research to identify subgroups with distinct characteristics that guide effective diagnosis, prognosis and treatment. This stratification can be performed based on molecular, genetic or clinical information. One such approach is to stratify patients based on the RNA expression of a certain gene of interest (GOI), which can serve to identify the effect of said gene in the biological processes involved in a certain disease. This has important implications for treatment decisions and improving our understanding of diseases.

However, this is a complex problem for which traditional bioinformatics methods are insufficient. A frequently employed approach is to simply rank samples by the GOI expression and compare the extremes of the distribution, by extracting for example the top and bottom 25% of samples [4]. This method neglects the underlying distribution of the data as well as other potentially relevant differentiating factors (i.e. functional profiles). More informed techniques include taking advantage of survival information [5, 6], which is not always available, or fitting two Gaussian functions to find the best separation point, which assumes a certain shape of the expression distribution [7].

We introduce GoiStrat, a workflow for the analysis of functional differences between low- and high-expressing groups of a GOI in any disease (Fig. 1). Phase I of this workflow is a novel, biologically-informed alternative approach to split RNA samples, whose goals are to maximise the biological differences between low- and high-expressing groups. This is based on a sliding-window algorithm that computes a functional enrichment score (FDS) based on GSVA matrices that capture the subtle variations in the activity of biological pathways. Phase II is a comprehensive (multi)-omics analysis of the functional differences between these groups, which includes differential analyses, PPI network construction, protein embedding and clustering, and functional profiling of the GOI and its protein community.

We compared the ability of GoiStrat and the top/bottom approach in separating samples based on functional differences in 8 TCGA RNA-Seq datasets. Our results showed that GoiStrat outperformed the top/bottom approach in all datasets except one, achieving the maximum FDS. GoiStrat proved to be a better alternative to separate samples such that the changes observed in biological processes due to varying expression of a GOI can be better studied.

We also present a full use case of our workflow with a dataset of nearly a thousand PCa samples (i.e. PCTA-WCDT) to investigate the biological role of *FOLH1* in PCa, yielding results in accordance with the literature. To the best of our knowledge, this approach has not yet been employed to identify the biological role of a known biomarker in PCa. Through the proposed method, in the multi-omics differential analyses between low- and high-expressing *FOLH1* groups, we identified related pathways such as the PI3K-AKT-mTOR gene sets as well as signatures linked to tumour aggressiveness, such as cholesterol homeostasis and apoptosis, among many others [12]. Furthermore, our method demonstrated a positive correlation between increased expression levels of *FOLH1* and tumourigenesis in both primary and metastatic samples. Thanks to each stage of our workflow, from the identification of key genes to the machine learning based clustering of protein communities, we were able to successively narrow down the functional profile of *FOLH1*. Therefore, we concluded that our methodology was able to successfully discover biological differences due to distinct expression levels of a certain GOI.

In this study we focused on RNA-Seq as well as methylation data due to the public availability of hundreds of samples, which significantly strengthened our findings. Integrating these two types of data enabled a more in-depth biological interpretation. Thus, we were able to understand the underlying epigenetic changes that contribute to RNA expression patterns, such as IL6-JAK-STAT3 signalling pathway in cancer patients. This is poised to provide therapeutic benefit by directly inhibiting tumour cell growth and by stimulating antitumour immunity [60]. These findings can offer valuable diagnostic or prognostic information.

However, translating discoveries made from RNA and methylation data into clinical settings is a very challenging endeavour. While some studies suggest that highly differentially expressed genes are more likely correlated with their gene products [84], the general consensus is that there is a discrepancy between RNA expression and protein expression due to post-transcriptional mechanisms [85] or delayed synthesis [86]. As a result, while our workflow provides a multitude of biological pathways and potential targets worthy of investigation, their utility as clinical targets will need further investigation.

As with any other method, our workflow comes with its own limitations, mainly regarding the computational resources and the biological interpretation of the results. Several steps of our workflow are computationally demanding and require significant computing resources. For example, the computation of the FDS is done for hundreds of iterations of the sliding window algorithm, which grows linearly with the number of samples. The z-score normalisation of the relative gene occurrence to select the top genes from the up-regulated gene sets, as well as the PPI clustering to identify the PSMA clusters also require considerable computing power. Our workflow was implemented in Python and took advantage of multi-processing to speed up computations whenever possible. The code implementation was designed with flexibility, extensibility and ease-of-use in mind, and special care was taken to keep all modules thoroughly documented.

Finally, it’s important to highlight the difficulty in evaluating functional differences between groups of samples purely based on RNA expression data and in-silico experiments. While we believe that FDS is a reasonable score to assess biological differences, we recognise that it is solely based on one single sequencing data type and an incomplete database of gene sets (i.e. MSigDB). Therefore, in order to truly assess biological differences due to the varying expression of a GOI, it is most likely inevitable to reinforce GoiStrat with wet-lab experiments.

## Conclusions

To summarise, patient stratification has revolutionized cancer prognosis and diagnostic approaches by acknowledging and leveraging the inherent diversity among patients. Through the identification of molecular subtypes, genetic markers, and immune profiles, stratification not only improves the precision of diagnosis and prognostication but also opens new avenues for personalised treatment strategies. As cancer research continues to evolve, stratification will remain a cornerstone of efforts to optimize therapeutic outcomes and improve patient care.

Our integrated bioinformatics approach provides a novel tool to identify disease-relevant functions of GOIs in large datasets to study their role for disease relevant pathways and to identify associated protein networks. This provides a basis for hypothesis building and subsequent functional analysis of target genes and might be highly relevant for biomarker identification or development of personalised therapies.

## Supplementary Information

Additional file 1.

Additional file 2.

## Data Availability

The datasets underlying this article include publicly available sources such as TCGA-PRAD [29] and PCTA [28], as well as WCDT-MCRPC [30, 31], whose access was granted upon permission for this research. All relevant data generated during the analysis and presented in the figures are available in the supplementary material (i.e. Additional files 1-4). The source code of this workflow is available on https://github.com/CarlosUziel/goi-strat.
