# Supplementary material for: Goistrat: gene-of-interest-based sample stratification for the evaluation of functional differences
Source: BMC Bioinformatics. 2025 Apr 5;26:97. doi: 10.1186/s12859-025-06109-0 (PMC11971790; doi:10.1186/s12859-025-06109-0)
Supplement: Supplementary file 2 — Additional file 2. [file 12859_2025_6109_MOESM2_ESM.pdf]

Appendix A Supplementary Tables

Dataset	Group	GoiStrat	10/80/10	15/70/15	20/60/20	25/50/25	30/40/30
**PCTA-WCDT (FOLH1)**	**Low**	241	67	100	133	166	199
	**Mid**	331	528	462	396	330	264
	**High**	90	67	100	133	166	199
	**Total**	662	662	662	662	662	662
**TCGA-BRCA (BRCA1)**	**Low**	420	111	166	221	277	332
	**Mid**	553	884	774	664	552	442
	**High**	133	111	166	221	277	332
	**Total**	1106	1106	1106	1106	1106	1106
**TCGA-HNSC (TP63)**	**Low**	199	52	78	104	130	156
	**Mid**	260	416	364	312	260	208
	**High**	61	52	78	104	130	156
	**Total**	520	520	520	520	520	520
**TCGA-KIRC (CA9)**	**Low**	216	54	81	108	134	161
	**Mid**	268	429	375	321	269	215
	**High**	53	54	81	108	134	161
	**Total**	537	537	537	537	537	537
**TCGA-LGG (IDH1)**	**Low**	51	52	78	103	129	155
	**Mid**	258	412	360	310	258	206
	**High**	207	52	78	103	129	155
	**Total**	516	516	516	516	516	516
**TCGA-LUAD (NKX2-1)**	**Low**	212	53	80	106	132	159
	**Mid**	264	422	368	316	264	210
	**High**	52	53	80	106	132	159
	**Total**	528	528	528	528	528	528
**TCGA-LUSC (SOX2)**	**Low**	197	50	75	100	125	150
	**Mid**	250	401	351	301	251	201
	**High**	54	50	75	100	125	150
	**Total**	501	501	501	501	501	501
**TCGA-THCA (HMGA2)**	**Low**	51	51	76	101	126	152
	**Mid**	252	403	353	303	253	201
	**High**	202	51	76	101	126	152
	**Total**	505	505	505	505	505	505
**TCGA-UCEC (PIK3CA)**	**Low**	54	55	83	110	137	165
	**Mid**	274	439	383	329	275	219
	**High**	221	55	83	110	137	165
	**Total**	549	549	549	549	549	549

Table A1: Group sizes after multiple splitting strategies. For each dataset and GOI, the number of samples in each group is shown for GoiStrat, as well as for all quantiles of the top/bottom strategy: 10% (10/80/10), 15% (15/70/15), 20% (20/60/20), 25% (25/50/25) and 30% (30/40/30).

Dataset	GoiStrat	10/80/10	15/70/15	20/60/20	25/50/25	30/40/30
**PCTA-WCDT (FOLH1)**	0.981038	0.819348	0.940427	0.982247	0.995088	0.998717
**TCGA-BRCA (BRCA1)**	0.998880	0.959824	0.995088	0.999489	1.000000	1.000000
**TCGA-HNSC (TP63)**	0.925594	0.714129	0.873482	0.948315	0.980114	0.992701
**TCGA-KIRC (CA9)**	0.901491	0.730564	0.885485	0.955221	0.982908	0.994008
**TCGA-LGG (IDH1)**	0.889976	0.714129	0.873482	0.946439	0.979351	0.992408
**TCGA-LUAD (NKX2-1)**	0.896017	0.722445	0.881602	0.951883	0.981561	0.993515
**TCGA-LUSC (SOX2)**	0.900175	0.696893	0.860368	0.940427	0.976009	0.990768
**TCGA-THCA (HMGA2)**	0.888462	0.705613	0.864867	0.942498	0.976890	0.991461
**TCGA-UCEC (PIK3CA)**	0.906953	0.738487	0.892908	0.958341	0.984753	0.994888

Table A2: Test power. For each dataset and GOI, the test power given the group splits is shown for GoiStrat, as well as for all quantiles of the top/bottom strategy: 10% (10/80/10), 15% (15/70/15), 20% (20/60/20), 25% (25/50/25) and 30% (30/40/30).

MSigDB Category	Enrichment Level	Primary high vs. low			Metastatic high vs. low		
		D-GSVA	GSEA DE	GSEA DM	D-GSVA	GSEA DE	GSEA DM
**C1**	**down**	81	7	6	14	6	4
	**up**	70	3	4	38	0	10
**C2**	**down**	2379	472	24	1945	391	4
	**up**	2114	208	362	622	16	4
**C3**	**down**	639	35	2	283	10	0
	**up**	834	4	122	1379	0	0
**C4**	**down**	324	174	29	348	205	1
	**up**	388	109	59	128	9	0
**C5**	**down**	5256	815	22	3566	535	0
	**up**	5766	160	266	1793	16	6
**C6**	**down**	124	61	0	124	80	4
	**up**	28	0	50	10	0	0
**C7**	**down**	1588	143	0	1166	131	0
	**up**	2027	0	168	661	0	0
**C8**	**down**	426	218	3	252	182	2
	**up**	117	10	154	46	0	2
**H**	**down**	19	12	1	20	11	0
	**up**	24	5	14	4	0	0

Table A3: Number of differentially enriched gene sets for each MSigDB category, enrichment method and enrichment level of the gene sets from which the genes/proteins were extracted.

Cluster ID	Primary		Metastatic	
	up-regulated	down-regulated	up-regulated	down-regulated
**0**	794	2425	936	538
**1**	1172	469	35	17
**2**	901	532	93	164
**3**	1483	992	116	180
**4**	1936	535	360	516
**5**	0	494	275	452
**6**	0	0	489	498
**7**	0	0	331	783
**8**	0	0	415	125
**9**	0	0	523	0
**10**	0	0	176	0

Table A4: Size of clusters after ensemble clustering using Node2Vec embeddings, per sample type and enrichment level of the gene sets from which the genes/proteins were extracted.

Appendix B Supplementary Figures

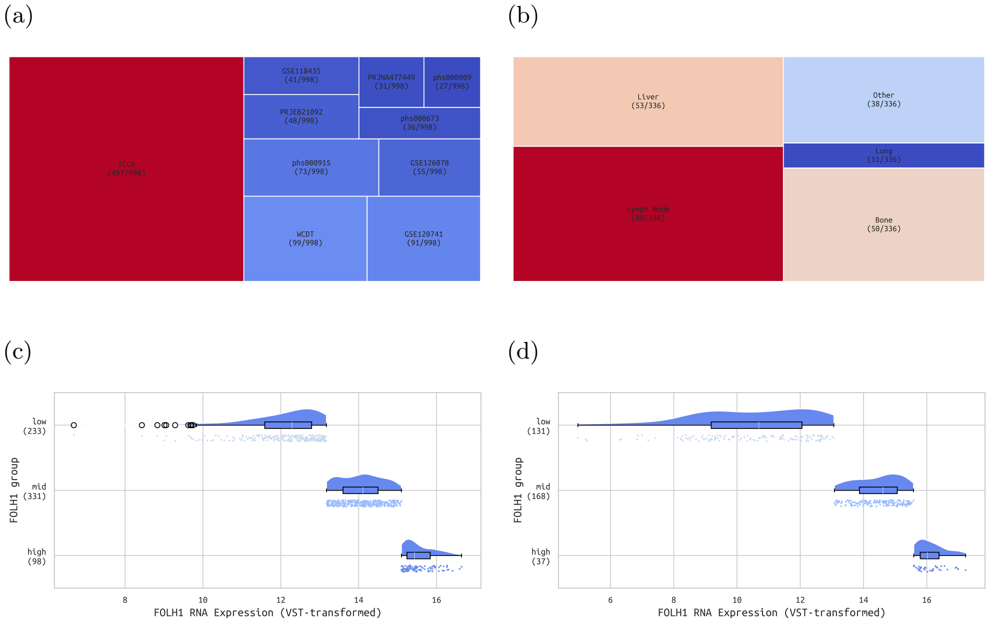

Fig. B1: **Data processing and sample stratification**. (a) PCTA-WCDT dataset composition. (b) Tissue of origin of metastatic samples in PCTA-WCDT. *FOLH1* RNA expression distribution of stratified PCTA-WCDT in (c) primary tumour samples and (d) metastatic tumour samples. See source data in Additional file 1.

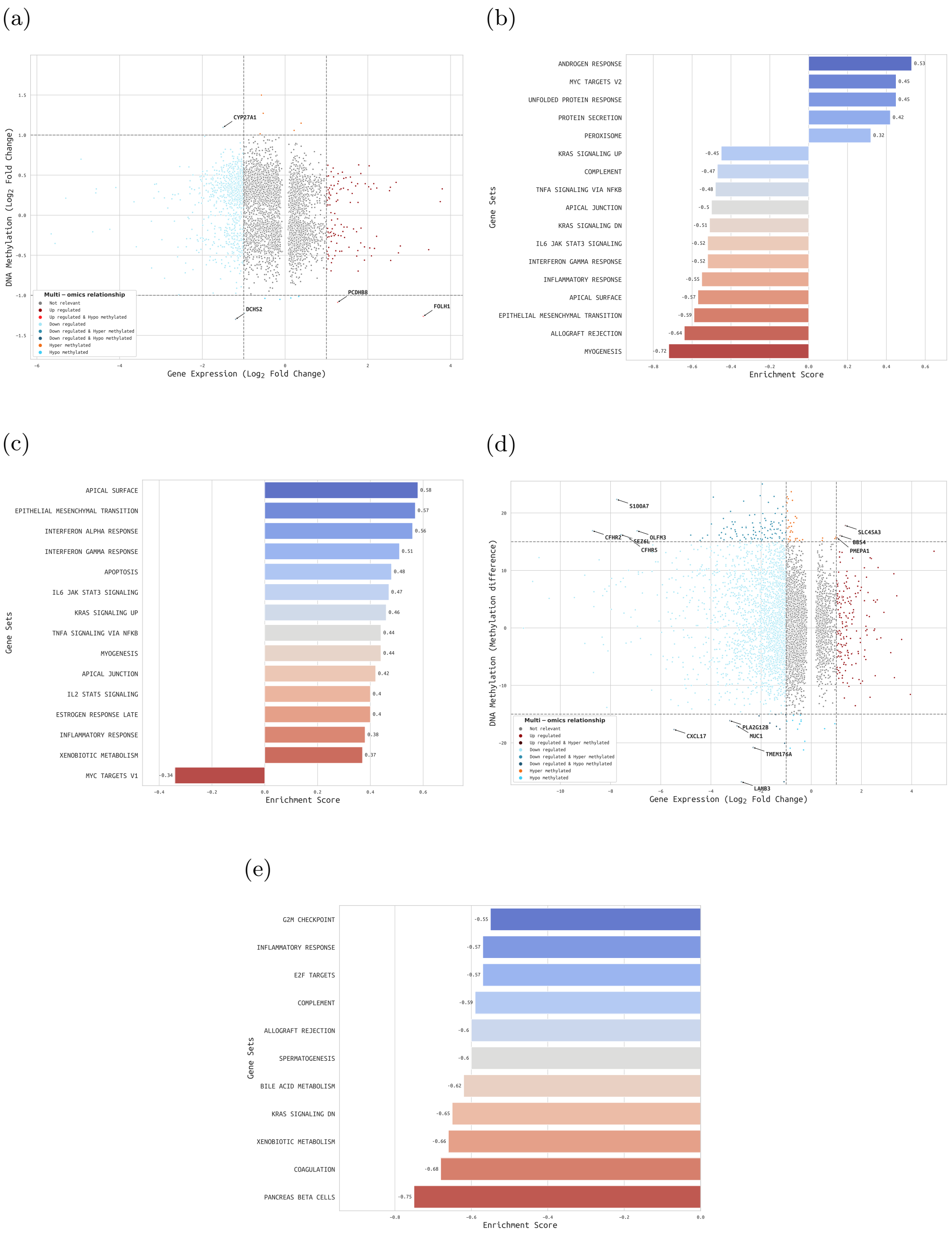

Fig. B2: **Differential Analyses**. (a) A starburst plot showing the relationship between the Log_2 fold changes from differential methylation and differential gene expression analyses in primary samples. up-regulated gene sets from the MSigDB Hallmarks collection after applying GSEA on (b) DEGs and (c) DMRs from primary samples. (d) A starburst plot showing the relationship between the Log_2 fold changes from differential methylation and differential gene expression analyses in metastatic samples. (e) up-regulated gene sets from the MSigDB Hallmarks collection after applying GSEA on DEGs from metastatic samples. See source data in Additional file 2.
